# Supplementary material for: Characterizing epidemiology of prediabetes, diabetes, and hypertension in Qataris: A cross-sectional study
Source: PLoS One. 2021 Oct 26;16(10):e0259152. doi: 10.1371/journal.pone.0259152 (PMC8547702; doi:10.1371/journal.pone.0259152)

**S2 Fig**. Age-distribution of prediabetes mellitus, diabetes mellitus, and hypertension among Qataris aged 18-64 years old


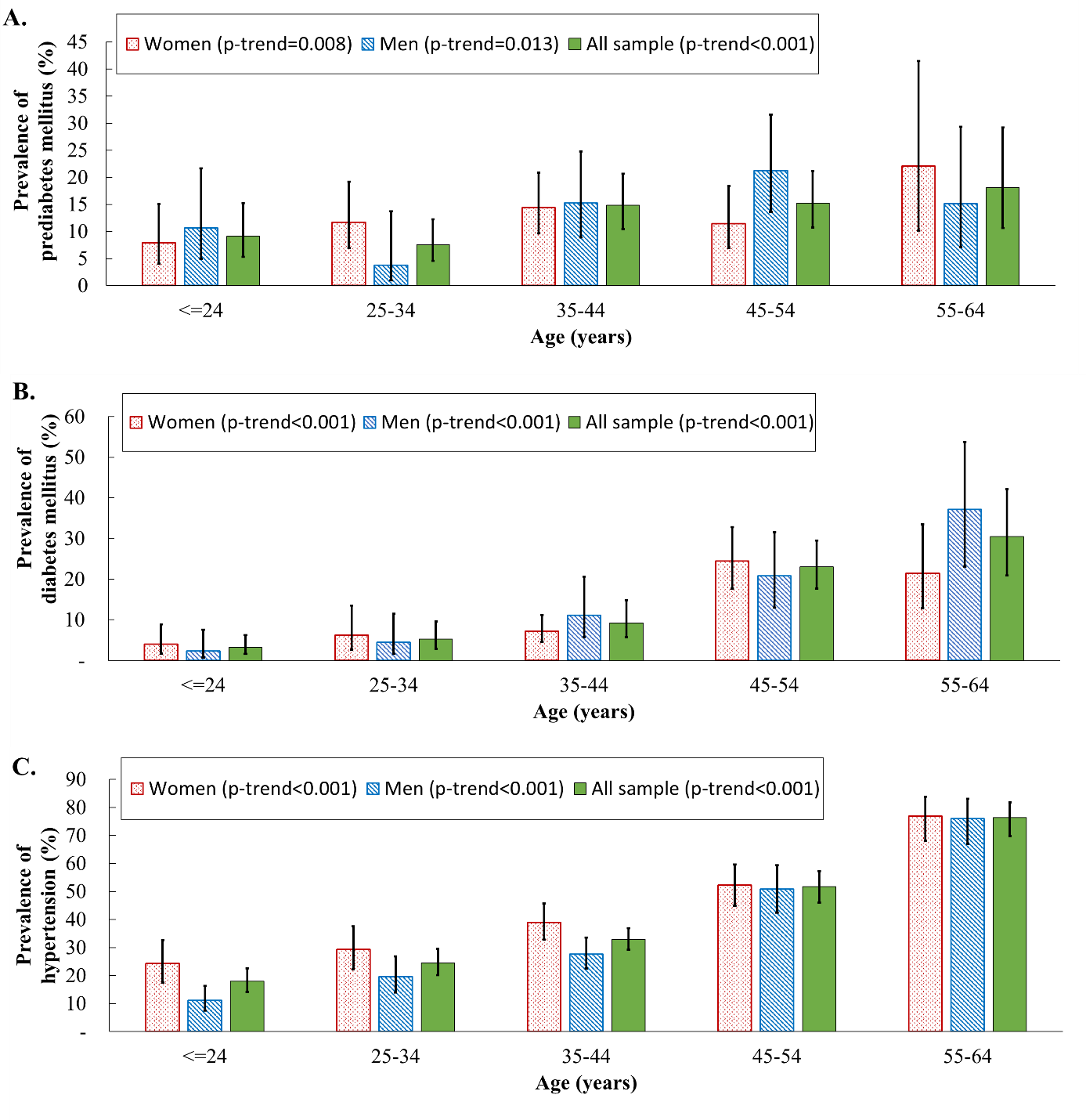

Supplement: S2 Fig — (DOCX) [file pone.0259152.s002.docx]
