# Supplementary material for: Characterizing epidemiology of prediabetes, diabetes, and hypertension in Qataris: A cross-sectional study
Source: PLoS One. 2021 Oct 26;16(10):e0259152. doi: 10.1371/journal.pone.0259152 (PMC8547702; doi:10.1371/journal.pone.0259152)
Supplement: S1 Appendix — (PDF) [file pone.0259152.s003.pdf]

PS002580E.Rev.3.qxp_PS-002580E R3 1005 Glu 11/12/14 5:43 PM Page 1

PS-002580E Rev. 3 10/05

PTS PANELS™ Glucose Test Strips

for use with CardioChek™ Brand Analyzers

INTENDED USE
The PTS PANELS Glucose Test Strips are intended to be used by healthcare professionals to measure glucose in whole blood and by individuals with diabetes to measure glucose in finger-stick whole blood at home. Glucose measurements are used in the management of carbohydrate metabolism disorders.

SUMMARY
Glucose is a sugar that is the major energy source in the body. Maintaining appropriate glucose levels is very important. This system may be used to monitor glucose. A MEMo Chip™ is provided with each package of test strips and must be properly inserted into the analyzer before any test can be run. The MEMo Chip contains test name, calibration curve, lot number and test strip expi-ration date. After the test strip is inserted into the analyzer and blood applied to the strip, test results are displayed in about a minute.

PRINCIPLES OF THE TEST
Glucose test results are based on the analyzer reading light reflected off a test strip that has changed color after blood has been placed on it. The darker the color, the higher the glucose level. The analyzer converts this reading into a glucose result and displays the results.

MATERIALS PROVIDED
- PTS PANELS Glucose Test Strips
- MEMo Chip (contains lot-specific test strip information)
- Instructions.

MATERIALS NEEDED BUT NOT PROVIDED
- CardioChek brand analyzer
- Quality Control Materials
- Lancets for fingerstick (or venous blood collection supplies)
- Alcohol wipes and/or gauze
- Capillary Blood Collector or other precision pipet for blood collection and application

CHEMICAL COMPOSITION
Each Glucose Test Strip contains the following active ingredients:
Glucose oxidase (Aspergillus niger) . . . . $\geq$ 0.2 I.U.
Peroxidase (Horseradish) . . . . $\geq$ 0.2 I.U.
4-aminoantipyrine . . . . $\geq$ 10 µg
N,N-disubstituted aniline . . . . $\geq$ 20 µg
Each vial contains not more than 5g silica gel desiccant.

STORAGE AND HANDLING
- Store test strip package in a cool, dry place at room temperature of 68-86°F (20-30°C). Strips may be stored in a refrigerator at 35-46°F (2-8°C), but must be brought to room temperature before using. Do not freeze.
- Keep away from heat and direct sunlight.
- Do not remove or discard the desiccant packet in the vial.
- Always replace vial cap immediately after removing a test strip.
- Use test strip as soon as you have removed it from the vial.
- Keep the MEMo Chip either in the analyzer or stored with the original lot of strips.
- Store the test strips in the original vial. Do not combine with other strips and do not store the MEMo Chip in the test strip vial.
- After opening, the test strips are stable until expiration date if vial is properly stored and always capped.

PRECAUTIONS
- For *in vitro* diagnostic use. Intended for self-testing.
- PTS PANELS Test Strips can only be used in the CardioChek brand analyzer.
- Make sure the MEMO Chip and test strip lot numbers match. Never use a MEMo Chip from a different lot than the test strip.
- Out-of-date or expired strips cannot be used in your test system. Check vial for expiration date.
- Add all of the blood to the test strip at once. If you do not get all of the blood on the strip, do not add blood to the same strip. Test again with a new unused test strip and fresh blood sample.
- Discard test strip after using. Strips are to be read once. Never insert or read a used test strip.
- **Do not ingest.**

SPECIMEN COLLECTION AND PREPARATION
PTS PANELS Test Strips are designed for use with fresh capillary (fingerstick) whole blood. Fresh venous whole blood collected in EDTA or heparin tubes is also an acceptable sample. To obtain a drop of blood from a fingerstick, follow the steps below:
- Use of lotions and handcreams should be avoided before testing.
- Hands should be washed in warm water with antibacterial soap, rinsed and dried thoroughly.
- If you wipe the fingertip with alcohol, be sure that the alcohol dries completely before sticking the finger.
- Use a sterile, disposable lancet to puncture the side of the fingertip.
- Wipe away the first drop of blood with a clean piece of gauze.
- Gently, without force, apply pressure to the fingertip to accumulate a drop of blood.
- Excessive squeezing of the finger may alter test results.
- See the "TESTING" section for information on how to apply the blood to the test strip.
- Discard used materials properly.

Caution: Handle and dispose of all materials coming in contact with blood according to universal precautions and guidelines.

TESTING
IMPORTANT: Read all instructions carefully before testing.

1. Insert the MEMo Chip that matches the lot number on the test strip vial and press one of the buttons to turn the analyzer ON.

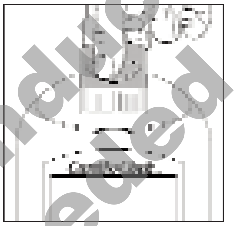

2.* Hold the test strip by the end with the horizontal raised lines. Insert the opposite end of the strip into analyzer. Push the strip in as far as it will go.

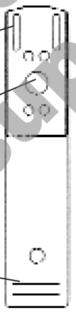

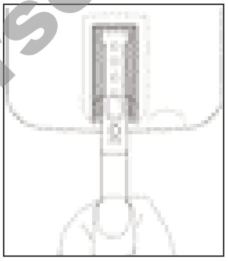

3. When APPLY SAMPLE appears on the display, use a capillary blood collector or pipet to apply 15 µL of whole blood to the test strip blood application window.

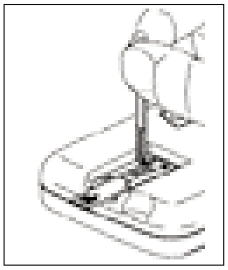

4. In about a minute, the result will appear on the display. Remove and discard strip. DO NOT add more blood to a test strip that has been used.

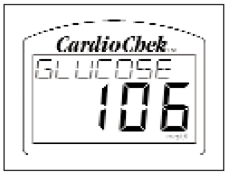

* As an alternative, the test strip may be inserted into the analyzer within 10 seconds AFTER blood is applied to the strip, when blood is applied to the strip directly from a finger. Touch a drop of blood hanging from the finger to the blood application window of the test strip. The blood drop must fill the entire window. Insert the strip into the analyzer. In about a minute, read result.

ADDITIONAL CONSIDERATIONS
1. If no result is displayed, make sure:
   – Enough blood was added to the test strip to completely fill the blood application window.
   – Analyzer is ON. (If it won't turn ON, refer to analyzer User Guide section on changing batteries.)
   – MEMo Chip is properly installed in port.
2. If you get a reading of "LOW", "<___", "HIGH", ">___" or any unexpected result, test again.
3. See analyzer User Guide Troubleshooting section for additional help.
4. To verify enough blood has been applied to the test strip, remove strip after testing and check back side of reaction area. Reaction area should be completely and evenly colored. If the area is not completely and evenly colored, discard the used test strip and test again.

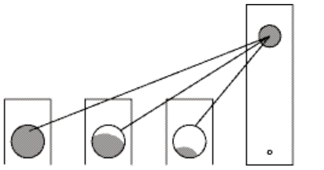

Manufactured by
Polymer Technology Systems, Inc.
Indianapolis, IN 46268 USA
CUSTOMER SERVICE (877)870-5610 (toll-free inside the U.S.)
(317)870-5610 Fax (317)870-5608

This insert was valid at the time of the conduct of the survey, but has since been superseded

TEST RESULTS
Results are displayed in either milligrams per deciliter (mg/dL) or in millimoles per liter (mmol/L). The mg/dL measurement is a US version, while mmol/L is used in many countries around the world. The analyzer is preset to US units by the manufacturer. No calculation of results are necessary. To change to INTL (mmol/L) units, please see the analyzer User Guide.

QUALITY CONTROL
Please refer to the analyzer User Guide for the proper procedure and materials to be used to perform Quality Control tests. Quality Control tests are used to ensure that the system (analyzer, strips and MEMo Chip) is working properly. Users should run controls when results are questionable or to comply with their own facility's quality control requirements.

EXPECTED VALUES
Blood glucose levels will vary from time to time depending on food consumed, activity levels, health status, medication dosages, stress or exercise. Your physician or healthcare professional will discuss "target values" (that is, highs and lows) specifically appropriate for you. A glucose level below 50 mg/dL (2.78 mmol/L) or above 240 mg/dL (13.32 mmol/L) may indicate a serious medical condition. If your test result should fall below 50 mg/dL (2.78 mmol/L) or exceed 240 mg/dL (13.32 mmol/L), you should contact your physician or healthcare professional as soon as possible.
Expected values are for a fasting person, who does not have diabetes are: 70-105 mg/dL (3.9-5.8 mmol/L).[6]

MEASURING RANGE
The glucose test system will detect glucose levels from 20-600 mg/dL (1.11-33.3 mmol/L) and will display a number value for results in this range. If the display reads "LOW" or "<___" (less than measuring range), the glucose level is below 20 mg/dL (1.11 mmol/L). Results above 600 mg/dL (33.3 mmol/L) will read "HIGH" or ">___" (greater than measuring range).
The analyzer will display "CHECK KETONE" for glucose test results greater than 240 mg/dL (13.32 mmol/L).
IMPORTANT: If a "LOW", "HIGH", "<" or ">" or any unexpected result is displayed, always test again with a new, unused test strip.

LIMITATIONS OF THE PROCEDURE
1. PRESERVATIVES: Blood samples preserved with Fluoride or Oxalate should not be used for testing with this system.
2. NEONATAL USE: This product has not been tested using neonatal blood. Until testing is done, this test system should not be used on neonatal blood samples.
3. METABOLITES: This test system is specific for glucose. Other sugars and other reducing substances such as Vitamin C at normal blood concentrations have no significant effect on test results.
4. HEMATOCRIT: Hematocrit values above 55% or lower than 30% may incorrectly lower the glucose result.
5. ALTITUDE: Testing at altitudes up to 5280 feet has no effect on results.
6. DEHYDRATION: Severe dehydration and excessive water loss may produce falsely low results.

PERFORMANCE CHARACTERISTICS
1. ACCURACY: PTS PANELS Glucose Test strip results are calibrated to provide plasma glucose values. The Glucose Test Strips were calibrated to an automated glucose hexokinase laboratory method run on plasma samples. In a method comparison to a leading commercially available glucose system (a biosensor glucose dehydrogenase method) that is calibrated to provide "plasma-like" values[5], the results below show that the PTS PANELS Glucose Test Strips compare well. This means that the PTS PANELS Glucose Test Strips should compare well to a laboratory plasma method:
   PTS PANELS Glucose vs. Commercially Available Glucose System
   Number of patients = 120
   slope = 0.951
   y-intercept = 5.36
   r = 0.99
   Previously, Glucose Test Strips were calibrated to provide whole blood glucose values. In a patient use clinical study performed in a diabetes clinic, Glucose Test Strip patient-run test results from fresh capillary blood specimens were compared to the results from the same specimens run by a professional on a Yellow Springs Instruments (YSI) Glucose Analyzer:
   PTS PANELS Glucose vs. YSI Glucose
   Number of patients = 86
   slope = 0.99
   y-intercept = 4.63
   r = 0.978
   A professional patient study of 161 patients performed by healthcare professionals at three different sites gave the following results:
   PTS PANELS Glucose vs. YSI Glucose
   Number of patients = 161
   slope = 0.983
   y-intercept = 0.67
   r = 0.97
2. PRECISION: Twenty replicates of various levels of whole blood were tested for glucose. The following results were obtained:

No. of Samples	20	20	20	20	20
Mean Glucose Conc. (mg/dL)	41	87	104	197	368
Std. Deviation (mg/dL)	2.75	4.66	5.90	5.24	13.69
Coefficient of Variation (%)	6.67	5.35	5.68	2.67	3.72

   This means that the variation between test strips is not greater than 6.7%.
3. INTERFERENCES: See LIMITATIONS section.

AVAILABILITY

REF/CAT NO.	DESCRIPTION
1713	PTS PANELS Glucose Test Strips – 25 Tests
0721	PTS PANELS Multi-Chemistry Controls – Level 1 & Level 2
1709	CardioChek Analyzer
1708	CardioChek P•A Analyzer

CLIA INFORMATION (US only)
Complexity Categorization: Waived

REFERENCES

1. Data on file, Polymer Technology Systems, Inc., Indianapolis, IN 46268.
2. Clinical Diagnosis and Management by Laboratory Methods, Eighteenth Edition, John Bernard Henry, Editor,. W.B. Saunders Company, Philadelphia, 1991.
3. NCCLS Proposed Guideline EP6-P, Evaluation of the Linearity of Quantitative Analytical Methods. Villanova, PA: National Committee for Clinical Laboratory Standards, 1986.
4. NCCLS Tentative Guideline EP7-T. Interference Testing in Clinical Chemistry. Villanova, PA: National Committee for Clinical Laboratory Standards, 1986.
5. Accu-Chek Comfort Curve instructions, Roche Diagnostics, 2000.
6. Clinical Chemistry, Third Edition, Norbert W. Tietz, Ph.D., Editor, W.B. Saunders Company, Philadelphia, 1987.

CUSTOMER SERVICE
Customer Service is available to answer questions regarding the CardioChek brand analyzers and PTS PANELS Test Strips. Outside Customer Service hours, please contact your healthcare professional.

(877) 870-5610 (8 a.m. – 5 p.m. EST, M-F toll-free inside the USA)
(317) 870-5610, FAX 1 (317) 870-5608
E-mail inforequest@cardiochek.com

The CardioChek brand analyzers and PTS PANELS Test Strips are manufactured in the US by Polymer Technology Systems, Inc., Indianapolis, IN 46268.

Copyright © 2005 by Polymer Technology Systems, Inc.

CE 0197

AUTHORIZED EUROPEAN REPRESENTATIVE
per IVDD 98/79/EC
MDSS GmbH
D-30163 Hannover
Germany

Explanation of Symbols

Symbol	Meaning	Symbol	Meaning
(hourglass)	Use By/ Expiration date	REF	Catalog number
LOT	Batch Code/ Lot number	(book)	Consult instructions for use
IVD	For in vitro diagnostic use	(factory)	Manufacturer
CE 0197	This product fulfills the requirements of Directive 98/79/EC on in vitro diagnostic medical devices.	(thermometer)	Store at/Temperature limitation

This insert was valid at the time of the product
in the survey, but has since been superseded
