# Supplementary material for: Characterizing epidemiology of prediabetes, diabetes, and hypertension in Qataris: A cross-sectional study
Source: PLoS One. 2021 Oct 26;16(10):e0259152. doi: 10.1371/journal.pone.0259152 (PMC8547702; doi:10.1371/journal.pone.0259152)
Supplement: S2 Appendix — (PDF) [file pone.0259152.s004.pdf]

S2 Appendix
English version of the “National STEP wise Survey of Chronic Non-communicable Diseases & Risk Factors Instrument”

National STEP wise Survey of Chronic Non-communicable Diseases & Risk Factors Instrument

(Core and Expanded)

National STEP wise Survey
of Chronic Non-communicable Disease s
& Risk Factor

Survey Information

	Location and Date	Response	Code
1	Municipality No.	|__|__|	I1
2	Area No.	|__|__|	I2
3	Census square No.	|__|__|__|	X1
4	Cluster No.	|__|__|__|__|	X2
5	Electricity No.	|__|__|__|__|__|__|__|	X3
6	Interviewer ID	|__|__|__|	I3
7	Date of completion of the instrument	|__|__| |__|__| |__|__|__|__| dd mm year	I4

✂ -- ✂

Participant Id Number \|__\|__\|__\| \|__\|__\|__\| \|__\|__\|__\|

	Consent, and Name	Response	Code
8	Consent has been read and obtained	Yes 1<br>No 2 **If NO, END**	I5
9	Time of interview<br>(24 hour clock)	|__|__| : |__|__| hrs mins	I7
10	Family Surname		I8
11	First Name		I9
	**Additional Information that may be helpful**		
12	Contact phone number where possible (mobile, home; work)		I10

Record and file identification information (I5 to I10) separately from the completed questionnaire.

Step 1 Demographic Information

CORE: Demographic Information

Question		Response	Code
13	Sex (*Record Male / Female as observed)*	Male 1<br>Female 2	C1
14	What is your date of birth?<br>*Don't Know 77 77 7777*	└─┴─┘ └─┴─┘ └─┴─┴─┴─┘ *If known, Go to C4*<br>dd mm year	C2
15	How old are you?	Years └─┴─┘	C3
16	In total, how many years have you spent at school or in full-time study (excluding pre-school)?	Years └─┴─┘	C4

EXPANDED: Demographic Information

17	What is the **highest level of education** you have completed?	No formal schooling 1<br>Less than primary school 2<br>Primary school completed 3<br>Preparatory school completed 4<br>Secondary school completed 5<br>College/University completed 6<br>Post graduate degree 7<br>Refused 88	C5
18	What is your **marital status**?	Never married 1<br>Currently married 2<br>Divorced 3<br>Widowed 4<br>Refused 88	C7
19	Which of the following best describes your **main work** status over the past 12 months?	Government employee 1<br>Non-government employee 2<br>Self-employed 3<br>Non-paid 4<br>Student 5<br>Homemaker 6<br>Retired 7<br>Unemployed (able to work) 8<br>Unemployed (unable to work) 9<br>Satisfied (Doesn't work and not looking for work) 10<br>Refused 88	C8

20	Is there a family relationship (consanguinity) between the mother and father (parents)?	Yes 1<br>No 2	X4

Step 1 Behavioural Measurements

CORE: Tobacco Use

Now I am going to ask you some questions about various health behaviours. This includes things like smoking, eating fruits and vegetables and physical activity. Let's start with tobacco.

Question		Response	Code
21	Have **you ever smoked** any tobacco products, such as cigarettes, cigars or pipes or Shisha or Medwakh?<br>(USE SHOWCARD)	Yes 1<br>No 2 *If No, go to T9a*	T1a
22	Do you currently smoke any **tobacco products**, such as cigarettes, cigars, pipes or Shisha or Medwakh?<br>*(USE SHOWCARD)*	Yes 1<br>No 2 *If No, go to T6*	T1
23	Do you currently smoke tobacco products **daily**?	Yes 1<br>No 2 *If No, go to T6* a	T2
24	How old were you when you **first started** smoking daily?	Age (years)<br>Don't know 77 └─┴─┘ *If Known, go to T5a*	T3
25	Do you remember how long ago it was?<br>*(RECORD ONLY 1, NOT ALL 3)*<br>*Don't know 77*	In Years └─┴─┘ *If Known, go to T5a*	T4a
		OR in Months └─┴─┘ *If Known, go to T5a*	T4b
		OR in Weeks └─┴─┘	T4c
26	On average, **how many** of the following do you smoke each day?<br>*(RECORD FOR EACH TYPE, USE SHOWCARD)*<br>*Don't Know 77*	Manufactured cigarettes └─┴─┘	T5a
		Hand-rolled cigarettes └─┴─┘	T5b
		Pipes full of tobacco └─┴─┘	T5c
		Cigars, cheroots, cigarillos └─┴─┘	T5d
		Shisha └─┴─┘	X5
		Medwakh └─┴─┘	X6
		Other └─┴─┘ *If Other, go to T5other, else go to T6a*	T5e
		Other (please specify): └─┴─┴─┴─┴─┴─┘	T5other

27	During the past 12 months, have you tried to **stop smoking**?	Yes 1<br>No 2	T6a
28	During any visit of a doctor or other health worker in the past 12 months, were you advised to quit smoking tobacco?	Yes 1 *If T2=Yes, go to T9a*<br>No 2 *If T2=Yes, go to T9a*<br>No visit during the past 12 months 3 *If T2=Yes, go to T9a*	T6b

EXPANDED: Tobacco Use

Question		Response	Code
29	In the past, did you **ever** smoke **daily**?	Yes 1<br>No 2 *If No, go to T9a*	T6
30	How old were you when you **stopped** smoking **daily**?	Age (years)<br>Don't Know 77 └─┴─┘ *If Known, go to T9a*	T7
31	How **long ago** did you stop smoking daily?<br>*(RECORD ONLY 1, NOT ALL 3)*<br>*Don't Know 77*	Years ago └─┴─┘ *If Known, go to T9a*	T8a
		OR Months ago └─┴─┘ *If Known, go to T9a*	T8b
		OR Weeks ago └─┴─┘	T8c
32	Have you **ever** used **smokeless tobacco** products, such as [snuff, chewing tobacco, Swaika]? (USE SHOWCARD)	Yes 1<br>No 2 *If No, go to T13*	T9a
33	Do you **currently use** any **smokeless tobacco** such as *[snuff, chewing tobacco,* Swaika)? *(USE SHOWCARD)*	Yes 1<br>No 2 *If No, go to T12*	T9
34	Do you **currently use smokeless tobacco** products **daily?**	Yes 1<br>No 2 *If No, go to T12*	T10
35	On average, how many **times a day** do you use ….<br>*(RECORD FOR EACH TYPE, USE SHOWCARD)*<br>*Don't Know 77*	Snuff, by mouth └─┴─┘	T11a
		Snuff, by nose └─┴─┘	T11b
		Chewing tobacco └─┴─┘	T11c
		Swaika └─┴─┘	X7
		Other └─┴─┘ *If Other, go to T11other, else go to T13*	T11e
		Other (specify) └─┴─┴─┴─┴─┴─┴─┘ *Go to T13*	T11other
**36**	In the **past**, did you **ever use** smokeless tobacco such as *[snuff, chewing tobacco, or* Swaika,) **daily**?	Yes 1<br>No 2	T12
37	During the past 7 days, on how many days did someone **in your home** smoke when you were present?	Number of days<br>Don't know 77 └─┴─┘	T13
38		Number of days	T14

	During the past 7 days, on how many days did someone smoke in closed areas **in your workplace** (in the building, in a work area or a specific office) when you were present?	Don't know or don't work in a closed area 77 └─┴─┘	

**CORE: Diet**			
The next questions ask about the fruits and vegetables that you usually eat. I have a nutrition card here that shows you some examples of local fruits and vegetables. Each picture represents the size of a serving. As you answer these questions please think of a typical week in the last year.			
**Question**		**Response**	**Code**
39	How many days in the week, on do you **eat fruit**?<br>*(USE SHOWCARD)*	Number of days └─┴─┘ *If Zero days, go to D3*<br>Don't Know 77	D1
40	How many **servings** of fruit do you eat on one day? (*USE SHOWCARD)*	Number of servings<br>Don't Know 77 └─┴─┘	D2
41	How many days in the week do you **eat vegetables**? *(USE SHOWCARD)*	Number of days └─┴─┘ *If Zero days, go to D5*<br>Don't Know 77	D3
42	How many **servings** of vegetables do you eat on one day? *(USE SHOWCARD)*	Number of servings<br>Don't know 77 └─┴─┘	D4

**EXPANDED: Diet**			
43	What type of **oil or fat is most often** used for meal preparation in your household?<br><br>*(USE SHOWCARD)*<br>*(SELECT ONLY ONE)*	Vegetable oil 1<br>Vegetable ghee 2<br>Butter 3<br>Animal ghee 4<br>Other 5 *If Other, go to D5 other*<br>None in particular 6<br>None used 7<br>Don't know 77	D5
		Other └─┴─┴─┴─┴─┴─┴─┘	D5other

44	On average, how many meals per week do you eat that were not prepared at a home? By meal, I mean breakfast, lunch and dinner.	Number<br>Don't know 77 └─┴─┘	D6

**EXPANDED QATAR: Diet**			
45	In a typical **week**, on **how many days** do you eat or drink the following? *(USE SHOWCARD)*		
	Whole grain cereals and products (such as: brown bread, brown rice, oats, etc.)	Number of days<br>Don't Know 77 └─┴─┘	X8a
	Refined cereals and products (such as: white bread, rice, pasta, etc.)	Number of days<br>Don't Know 77 └─┴─┘	X8b
	Legumes (such as: beans, lentils, chickpeas, etc.)	Number of days<br>Don't Know 77 └─┴─┘	X8c
	Milk and dairy products (fresh milk, powder milk, yogurt, labneh, cheeses, eggs)	Number of days<br>Don't Know 77 └─┴─┘	X8d
	Fish and seafood	Number of days<br>Don't Know 77 └─┴─┘	X8e
	Poultry and chicken	Number of days<br>Don't Know 77 └─┴─┘	X8f
	lamb, beef	Number of days<br>Don't Know 77 └─┴─┘	X8g
	Sweets (such as: chocolate, candy, cake, baklawa, icecream, etc.)	Number of days<br>Don't Know 77 └─┴─┘	X8h
	Sugar sweetened beverages (such as: sodas (not diet), artificial juices, etc.)	Number of days<br>Don't Know 77 └─┴─┘	X8i

	Fresh fruit juices	Number of days<br>Don't Know 77 └─┴─┘	X8j
	Fast foods bought from a fast food restaurant (such as:<br>burgers fried chicken, shawarma, pizza, fries, pizza, etc.)	Number of days<br>Don't Know 77 └─┴─┘	X8k

CORE: Physical Activity

Now I am going to ask you about your physical activity during the week and the time you spend to exercise different types of physical activity. I hope to answer these questions even if you think you do not do any physical activity think first about physical activity at work, whether paid or unpaid, or household chores. (Insert other examples if needed) in answering the following questions, 'vigorous-intensity activities' can be defined as work that requires hard physical effort and cause large increase in breathing or heart rate. The 'moderate-intensity activities' is the activity that requires moderate physical effort and cause small increases in breathing or heart rate.

	Question	Response	Code
**Work**			
**46**	Does your **work involve vigorous-intensity** activity that causes large increases in breathing or heart rate like *[carrying or lifting heavy loads, digging or construction work]* for at least 10 minutes continuously?	Yes 1<br>No 2 *If No, go to P 4*	P1
**47**	How many days in a week, do you do vigorous-intensity activities as part of your work?	Number of days └─┘	P2
**48**	How much time do you spend doing vigorous-intensity activities at work on a day?	Hours : minutes └─┴─┘ : └─┴─┘ hrs mins	P3 (a-b)
**49**	Does your **work involve moderate-intensity** activity that causes small increases in breathing or heart rate such as brisk walking *[or carrying light loads]* for at least 10 minutes continuously?	Yes 1<br>No 2 *If No, go to P7*	P4
**50**	How many days in a week, do you do moderateintensity activities as part of your work?	Number of days └─┘	P5
**51**	How much time do you spend doing moderate-intensity activities at work on a typical day?	Hours : minutes └─┴─┘ : └─┴─┘ hrs mins	P6 (a-b)
**Travel to and from places**			
	The next questions exclude the physical activities at work t ıat you have already nentioned.<br>Now I would like to ask you about the usual way you travel *examples if needed]* ːo and from places. For ɜxample, f ır shopping, to market, to nosque. *[Insert other*		
**52**	Do you walk for at least 10 minutes continuously to get to and from places?	Yes 1<br>No 2 *If No, go to P 10*	P7
**53**	How many days in a week, do you walk for at least 10 minutes continuously to get to and from places?	Number of days └─┘	P8
**54**	How much time do you spend walking for travel on a day?	Hours : minutes └─┴─┘ : └─┴─┘ hrs mins	P9 (a-b)

CORE: Physical Activity, Continued

Question	Response	Code

**Recreational activities**			
The next questions exclude the work and transport activitie ; that you have already mentioned. Now I would like to ask you about sports, fitness and recrea tional activities (leisure), *[Insert relevant terms]*.			
55	Do you do any vigorous-intensity sports, fitness or recreational *(leisure)* activities that cause large increases in breathing or heart rate like *[running or football]* for at least 10 minutes continuously? *(USE SHOWCARD)*	Yes 1<br>No 2 *If No, go to P 13*	P10
56	How many days in a week do you do vigorous-intensity sports, fitness or recreational *(leisure)* activities?	Number of days └─┘	P11
**57**	How much time do you spend doing vigorous-intensity sports, fitness or recreational activities on a typical day?	Hours : minutes └─┴─┘ : └─┴─┘ hrs mins	P12 (a-b)
**58**	Do you do any moderate-intensity sports, fitness or recreational *(leisure)* activities that cause a small increase in breathing or heart rate such as brisk walking, *[cycling, swimming, volleyball]* for at least 10 minutes continuously? *(USE SHOWCARD)*	Yes 1<br>No 2 *If No, go to P16*	P13
59	How many days in a week, do you do moderateintensity sports, fitness or recreational *(leisure)* activities?	Number of days └─┘	P14
**60**	How much time do you spend doing moderate-intensity sports, fitness or recreational *(leisure)* activities on a day?	Hours : minutes └─┴─┘ : └─┴─┘ hrs mins	P15 (a-b)

**EXPANDED: Physical Activity**			
**Sedentary behaviour**			
The following question is about sitting or reclining at work, at home, getting to and from places, or with friends including time spent sitting at a desk, sitting with friends, traveling in car, reading, or watching television, but do not include time spent sleeping. *[INSERT EXAMPLES] (USE SHOWCARD)*			
61	In the previous week how much time do you spend **sitting or reclining on a day?**	Hours : minutes └─┴─┘ : └─┴─┘ hrs mins	P16 (a-b)
62	What is the total number of your daily working hours in a workweek?	Number of hours └─┴─┘	X9

**CORE: History of Raised Blood Pressure**		
**Question**	**Response**	**Code**

63	Have you ever had your blood pressure measured by a doctor or other health worker?	Yes 1<br>No 2 *If No, go to X10*	H1
64	Have you ever been told by a doctor or other health worker that you have raised blood pressure or hypertension?	Yes 1<br>No 2 *If No, go to X10*	H2a
65	Have you been told in the past 12 months?	Yes 1<br>No 2	H2b

**EXPANDED: History of Raised Blood Pressure**			
66	Are you currently receiving any of the following treatments/advice for high blood pressure prescribed by a doctor or other health worker?		
	Drugs (medication) that you have taken in the past two weeks	Yes 1<br>No 2	H3a
	Advice to reduce salt intake	Yes 1<br>No 2	H3b
	Advice or treatment to lose weight	Yes 1<br>No 2	H3c
	Advice or treatment to stop smoking	Yes 1<br>No 2	H3d
	Advice to start or do more exercise	Yes 1<br>No 2	H3e
67	Have you ever seen a traditional healer for raised blood pressure or hypertension?	Yes 1<br>No 2	H4
68	Are you currently taking any herbal or traditional remedy for your raised blood pressure?	Yes 1<br>No 2	H5
69	Is there a family history of hypertension or do one of your parents (father or mother), brothers or sisters, suffers from, high blood pressure (hypertension)?	Yes 1<br>No 2	X10

CORE: History of Diabetes

Question		Response	Code
70	Have you ever had your blood sugar measured by a doctor or other health worker?	Yes 1<br>No 2 *If No, go to* X13	H6
71	Have you ever been told by a doctor or other health worker that you have raised blood sugar or diabetes?	Yes 1<br>No 2 *If No, go to* X13	H7a
72	Have you been told in the past 12 months?	Yes 1<br>No 2	H7b

EXPANDED: History of Diabetes

73	Are you currently receiving any of the following treatments/advice for diabetes prescribed by a doctor or other health worker?		
	Insulin	Yes 1<br>No 2	H8a
	Drugs (medication) that you have taken in the past two weeks	Yes 1<br>No 2	H8b
	Special prescribed diet	Yes 1<br>No 2	H8c
	Advice or treatment to lose weight	Yes 1<br>No 2	H8d
	Advice or treatment to stop smoking	Yes 1<br>No 2	H8e
	Advice to start or do more exercise	Yes 1<br>No 2	H8f
74	Have you ever seen a traditional healer for diabetes or raised blood sugar?	Yes 1<br>No 2	H9
75	Are you currently taking any herbal or traditional remedy for your diabetes?	Yes 1<br>No 2	H10
76	Do you do testing for your blood sugar at home	Yes 1<br>No 2	X11
77	Do you visit the diabetes clinic or your doctor for diabetes on a regular basis?	Yes 1<br>No 2	X12

78	Is there a family history of diabetes, do your parents (father or mother) or children or brothers or sisters suffer from diabetes?	Yes 1<br>No 2	X13

**CORE: Oral health**			
The next questions ask about your oral health status and related behaviors			
**Questions**		**Response**	**Code**
79	How many natural teeth do you have?	No natural teeth 1 if no natural teeth, go to O3<br>1 to 9 teeth 2<br>10 to 19 teeth 3<br>20 teeth or more 4<br>Don't know 77	O1
80	How would you describe the **state of your teeth**?	Excellent 1<br>Very Good 2<br>Good 3<br>Average 4<br>Poor 5<br>Very Poor 6<br>Don't Know 77	O2
81	How would you describe the **state of your gums**?	Excellent 1<br>Very Good 2<br>Good 3<br>Average 4<br>Poor 5<br>Very Poor 6<br>Don't Know 77	O3
82	Do you have any **removable dentures**?	Yes 1<br>No 2 If No, go to O6	O4
83	Which of the following removable dentures do you have? (RECORD FOR EACH)		
	An upper jaw denture	Yes 1<br>No 2	O5a
	A lower jaw denture	Yes 1<br>No 2	O5b
84	During the past 12 months, did your teeth or mouth cause any **Pain or discomfort?**	Yes 1<br>No 2	O6

85	How long has it been since you last **saw a dentist**	Less than 6 months 1<br>6-12 months 2<br>More than 1 year but less than 2 years 3<br>2 or more years but less than 5 years 4<br>5 or more years 5<br>Never received dental care 6 If Never, go to O9	O7
86	What was the **main reason for your last visit** to the dentist?	Consultation / advice 1<br>Pain or trouble with teeth, gums or mouth 2<br>Treatment / Follow – up treatment 3<br>Routine check-up treatment 4<br>Other 5 If Other, go to O8 other	O8
		Other (please specify) └─┴─┴─┴─┴─┴─┘	O8other

CORE: Oral health, Continued

Questions		Response	Code
87	How **often do you clean** your teeth?	Never 1 If never, go to O13a<br>Once a month 2<br>2-3 times a month 3<br>Once a week 4<br>2-6 times a week 5<br>Once a day 6<br>Twice or more a day 7	O9
88	Do you use **toothpaste** to clean your teeth?	Yes 1<br>No 2 If No, go to O12a	O10
89	Do you use **toothpaste** containing **fluoride**?	Yes 1<br>No 2<br>Don't know 77	O11
90	Do you use any of the following to clean **your teeth**? (RECORD FOR EACH)		
	Toothbrush	Yes 1<br>No 2	O12a
	Wooden toothpicks	Yes 1<br>No 2	O12b
	Plastic toothpicks	Yes 1<br>No 2	O12c
	Thread (dental floss)	Yes 1<br>No 2	O12d
	Miswak	Yes 1<br>No 2	O12f
	Other	Yes 1 If Yes, go to O12other<br>No 2	O12g
	Other (please specify)	└─┴─┴─┴─┴─┴─┘	O12other
	Have you experienced **any of the following problems** during the past 12 months because of the **state of your teeth**? (RECORD FOR EACH)		

91	Difficulty in chewing foods	Yes 1<br>No 2	O13a
	Difficulty with speech / trouble pronouncing words	Yes 1<br>No 2	O13b
	Embarrassed about appearance of teeth	Yes 1<br>No 2	O13d
	Avoid smiling because of teeth	Yes 1<br>No 2	O13e
	Sleep is often interrupted	Yes 1<br>No 2	O13f
	Days not at work because of teeth or mouth	Yes 1<br>No 2	O13g
	Difficulty doing usual activities	Yes 1<br>No 2	O13h
	Reduced participation in social activities	Yes 1<br>No 2	O13j

**CORE: Injury**			
**The next questions ask about different experiences and behaviors that are related to road traffic injuries.**			
**Question**		**Response**	**Code**
92	In the past 30 days, how often did you use a seat belt when you were the driver or passenger of a motor vehicle?	All of the time 1<br>Sometimes 2<br>Never 3<br>Have not been in a vehicle in past 30 days 4<br>No seat belt in the car I usually am in 5<br>Don't Know 77<br>Refused 88	V1
**93**	In the past 30 days, how often did you wear a helmet when you drove or rode as a passenger on a motorcycle or motor-scooter?	All of the time 1<br>Sometimes 2<br>Never 3<br>Have not been on a motorcycle or motor-scooter in past 30 days 4<br>Do not have a helmet 5<br>Don't Know 77<br>Refused 88	V2
94	In the past 12 months, have you been involved in a road traffic crash as a driver, passenger, pedestrian, or cyclist?	Yes (as driver) 1<br>Yes (as passenger) 2<br>Yes (as pedestrian) 3<br>Yes (as a cyclist) 4<br>No 5 *If No, go toV5*<br>Don’t know 77 *If don't know, go to V5*<br>Refused 88 *If Refused, go to V5*	V3

**95**	Did you have any injuries in this road traffic crash which required medical attention?	Yes 1<br>No 2<br>Don't know 77<br>Refused 88	V4
**The next questions ask about the most serious accidental injury you have had in the past 12 months**			
96	In the past 12 months, were you injured accidentally, other than the road traffic crashes which required medical attention?	Yes 1<br>No 2 If No, go to step 2<br>Don't know 77 If don't know, go to step 2<br>Refused 88 If Refused, go to step 2	V5
97	Please indicate which of the following was the cause of this injury.	Fall 1<br>Burn 2<br>Poisoning 3<br>Cut 4<br>Near-drowning 5<br>Animal bite 6 Other (specify) 7<br>Don't know 77<br>Refused 88	V6
		Other (please specify) └─┴─┴─┴─┴─┴─┘	V6other
98	Where were you when you had this injury?	Home 1<br>School 2<br>Workplace 3<br>Road/Street/Highway 4<br>Farm 5<br>Sports/athletic area 6<br>Other (specify) 7<br>Don't know 77<br>Refused 88	V7
		Other (please specify) └─┴─┴─┴─┴─┴─┘	V7other

Step 2 Physical Measurements

**CORE: Height and Weight**			
**Question**		**Response**	**Code**
**99**	Interviewer ID	└─┴─┴─┘	M1
**100**	Device IDs for height and weight	Height └─┴─┘<br>Weight └─┴─┘	M2a<br>M2b

Participant Identification Number └─┴─┴─┘ └─┴─┴─┘ └─┴─┴─┘

**101**	Height	in Centimetres (cm) └─┴─┴─┘ . └─┘	M3
**102**	Weight<br>*If too large for scale 666.6*	in Kilograms (kg) └─┴─┴─┘ . └─┘	M4
**103**	**For women:** Are you pregnant?	Yes 1 *If Yes, go to M 8*<br>No 2	M5
**CORE: Waist**			
**104**	Device ID for waist	└─┴─┘	M6
**105**	Waist circumference	in Centimetres (cm) └─┴─┴─┘ . └─┘	M7
**106**	Interviewer ID	└─┴─┴─┘	M8
**107**	Device ID for blood pressure	└─┴─┘	M9
**108**	Cuff size used	Standard 1<br>Large 2	M10
109	Reading 1	Systolic (mmHg) └─┴─┴─┘	M11a
		Diastolic (mmHg) └─┴─┴─┘	M11b
**110**	Reading 2	Systolic (mmHg) └─┴─┴─┘	M12a
		Diastolic (mmHg) └─┴─┴─┘	M12b
111	Reading3	Systolic (mmHg) └─┴─┴─┘	M13a
		Diastolic (mmHg) └─┴─┴─┘	M13b
**112**	During the past two weeks, have you been treated for raised blood pressure with drugs (medication) prescribed by a doctor or other health worker?	Yes 1<br>No 2	M14

**EXPANDED: Hip Circumference and Heart Rat e**			
**113**	Hip circumference	in Centimeters (cm) └─┴─┴─┘ . └─┘	M15

Step 3 Biochemical Measurements

CORE: Blood Glucose

Question		Response	Code
114	During the past 12 hours have you had anything to e or drink, other than water? ıt	Yes 1<br>No 2	B1
115	Technician ID	|__|__|__|	B2
116	Device ID	|__|__|	B3
117	Time of day blood specimen taken (24 hour clock)	|__|__| : |__|__| Hours : minutes<br>hrs mins	B4
118	Fasting blood glucose	mg/dl |__|__|__| . |__|	B5
119	Today, have you taken insulin or other drugs (medication) that have been prescribed by a doctor or other health worker for raised blood glucose?	Yes 1<br>No 2	B6

CORE: Blood Lipids

120	Device ID	|__|__|	B7
121	Total cholesterol	mg/dl |__|__|__| . |__|	B8
122	During the past two weeks, have you been treated for raised by cholesterol with drugs (medication) prescribed a doctor or other health worker?	Yes 1<br>No 2	B9

EXPANDED: Triglycerides, HDL and LDL Cholesterol

123	Triglycerides	mg/dl |__|__|__| . |__|	B10
124	HDL Cholesterol	mg/dl |__|__|__| . |__|	B11
125	LDL Cholesterol	mg/dl |__|__|__| . |__|	X14

World Health Organization
