# Supplementary material for: Characterizing epidemiology of prediabetes, diabetes, and hypertension in Qataris: A cross-sectional study
Source: PLoS One. 2021 Oct 26;16(10):e0259152. doi: 10.1371/journal.pone.0259152 (PMC8547702; doi:10.1371/journal.pone.0259152)
Supplement: Attachment — Submitted filename: Review comments.docx [file pone.0259152.s005.docx]

The study looked at characterizing epidemiology of prediabetes, diabetes mellitus and hypertension in Qataris. The authors generated data from QATAR STEPWISE survey 2012. Major findings included increased prevalence of hyperglycemia and hypertension among the study participants. The authors did a tremendous work however, major and minor revisions are needed to improve the quality of the paper.

**Major revisions**

1. Results for several predictors were not sufficiently presented. There are no results for fasting blood glucose, blood pressure, waist/hip circumference and BMI. Though results for logistic regression exist, univariate analyses of these variables is very important

2. Blood glucose test was performed using POC with limited maximum cut off. The authors didn't write in their paper what was done to samples with blood glucose levels higher than what the device could read. Where they excluded from the statistical analysis? The statements under limitations on this was not sufficient

3. Discussion section was not thorough. The authors directed their findings to what should be done but did not excisively discuss it. For example lines 219-221 under the discussion section stated that the study under estimated prevalence of prediabetes and over estimated prevalence of diabetes mellitus and no clear explanation was provided for the difference

**Minor revisions**

1. I don't think I get the difference between p-trend and p-value, the authors may need to clarify this or be consistent since there was no mention of such under methods section

2. Lines 177, 186 and 197 mentioned "univariable"and I think it should be **univariate**

3. Lines 146 and 147 under results section has N=2497 or similar and since this represent samples it should be n=,.......

4. You stated prevalence of hyperglycemia rather than diabetes or prediabetes yet hyperglycemia is a state and not a disease and sometimes we have transferred hyperglycemia secondary to other non-diabetic causes. The authors may need to edit or revise that
